# Transcriptomic data analysis coupled with copy number aberrations reveals a blood-based 17-gene signature for diagnosis and prognosis of patients with colorectal cancer

**DOI:** 10.3389/fgene.2022.1031086

**Published:** 2023-01-06

**Authors:** Ibrahim H. Kaya, Olfat Al-Harazi, Dilek Colak

**Affiliations:** ^1^ College of Medicine, Alfaisal University, Riyadh, Saudi Arabia; ^2^ Department of Molecular Oncology, King Faisal Specialist Hospital and Research Centre, Riyadh, Saudi Arabia

**Keywords:** colorectal cancer, transcriptomic, genomic, biomarker, blood, gene signature, diagnosis, prognosis

## Abstract

**Background:** Colorectal cancer (CRC) is the third most common cancer and third leading cause of cancer-associated deaths worldwide. Diagnosing CRC patients reliably at an early and curable stage is of utmost importance to reduce the risk of mortality.

**Methods:** We identified global differentially expressed genes with copy number alterations in patients with CRC. We then identified genes that are also expressed in blood, which resulted in a blood-based gene signature. We validated the gene signature’s diagnostic and prognostic potential using independent datasets of gene expression profiling from over 800 CRC patients with detailed clinical data. Functional enrichment, gene interaction networks and pathway analyses were also performed.

**Results:** The analysis revealed a 17-gene signature that is expressed in blood and demonstrated that it has diagnostic potential. The 17-gene SVM classifier displayed 99 percent accuracy in predicting the patients with CRC. Moreover, we developed a prognostic model and defined a risk-score using 17-gene and validated that high risk score is strongly associated with poor disease outcome. The 17-gene signature predicted disease outcome independent of other clinical factors in the multivariate analysis (HR = 2.7, 95% CI = 1.3–5.3, *p* = 0.005). In addition, our gene network and pathway analyses revealed alterations in oxidative stress, STAT3, ERK/MAPK, interleukin and cytokine signaling pathways as well as potentially important hub genes, including *BCL2, MS4A1, SLC7A11, AURKA, IL6R, TP53, NUPR1*, *DICER1*, *DUSP5*, *SMAD3*, and *CCND1*.

**Conclusion:** Our results revealed alterations in various genes and cancer-related pathways that may be essential for CRC transformation. Moreover, our study highlights diagnostic and prognostic value of our gene signature as well as its potential use as a blood biomarker as a non-invasive diagnostic method. Integrated analysis transcriptomic data coupled with copy number aberrations may provide a reliable method to identify key biological programs associated with CRC and lead to improved diagnosis and therapeutic options.

## Introduction

Colorectal cancer (CRC) is the third most common cancer and the third highest cancer-related mortality worldwide (American Cancer Society, 2021; Sung et al., 2021). Despite all the advances in cancer therapies and raising awareness, colorectal cancer continues to be one of the deadliest cancers worldwide (Rawla et al., 2019; Xi and Xu, 2021). Diagnosing CRC patients during the early stages of the tumor development is essential, as that is when CRC is most curable. Therefore, it is of utmost importance to identify robust non-invasive diagnostic biomarkers for early detection of the cancer in order to achieve a better outcome. In addition, it is also essential to have biomarkers that would prognosticate patients with high-risk profiles to guide for personalized treatment.

Changes in gene expression and gene copy number are closely related to diseases such as cancer (Colak et al., 2010; Colak et al., 2013; Shao et al., 2019). Since tumorigenesis genes show associations with copy number variations (CNVs) and expression levels, it is possible to increase the diagnostic reliability as well as the predictive potential of prognosis by integrating CNV and gene expression data (Sheng et al., 2011; Miao et al., 2014; Shao et al., 2019; Kaya et al., 2022). Indeed, the previous studies, including our own, reported that the multi-omics approach may increase the robustness and reliability of biomarkers associated with complex diseases, including cancer (Miao et al., 2014; Aldosary et al., 2020; Das et al., 2020; Al-Harazi et al., 2021a; Baloni et al., 2021; Kaya et al., 2022; Ruan et al., 2022). Additionally, it has been reported that network-based approaches have high efficacy in identifying biomarkers for many complex diseases, including several different types of cancer (Wang et al., 2017; Chen et al., 2019; Liu et al., 2019; Uddin et al., 2019; Khan et al., 2020; Al-Harazi et al., 2021b). However, most biomarkers identified thus far require invasive procedures.

In this study, we identified a blood-based gene signature with diagnostic and prognostic potential for CRC by utilizing an integrated approach of transcriptomic analysis coupled with overlapping genes associated with the copy number alterations (CNA) in CRC. We then validated the gene signature’s classification performance as well as the prognostic potential using independent transcriptomics datasets from over 800 CRC patients with detailed clinical data. The identified gene signature may improve the diagnosis and prognosis of CRC and help to develop therapeutic strategies.

## Materials and methods

### Data collection and integrated analysis

**FIGURE 1:**
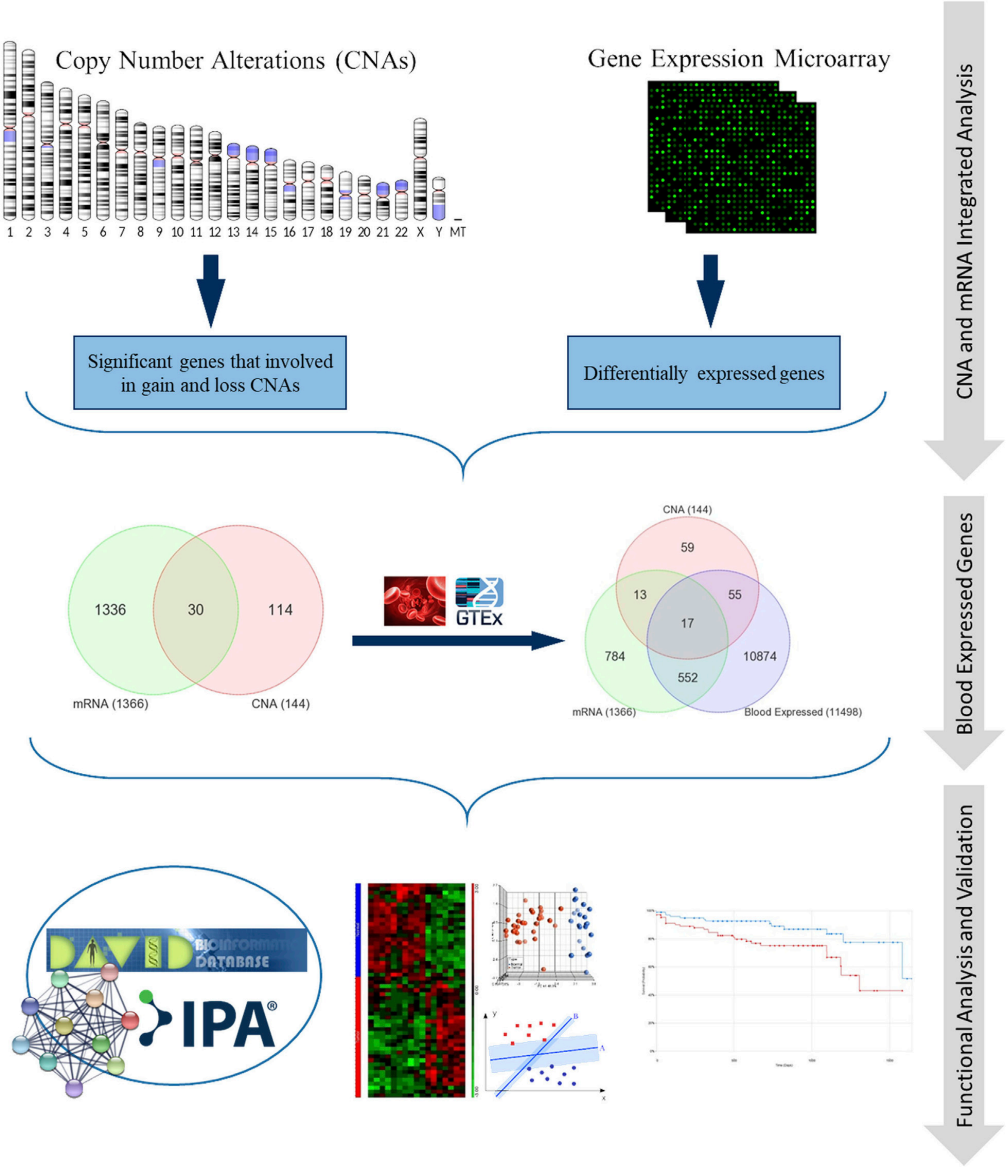
Schematic diagram illustrating the methodology.

Whole-genome gene expression dataset for patients with colorectal cancer (CRC) was gathered from GEO (GSE23878) (www.ncbi.nlm.nih.gov/geo). In addition, CNA regions associated with CRC in genomic data comprising thirty samples (15 tumor and 15 adjacent normal samples) from Saudi patients were identified as described previously in (Eldai et al., 2013). The gene expression dataset (GSE23878) contains samples from 35 colon tumors and 24 normal controls (Uddin et al., 2011). The samples were probed using Affymetrix Human Genome U133 Plus 2.0 Array. The differentially expressed genes (DEGs) were identified using independent two-sample *t*-test with adjusted *p*-value of <0.05 and absolute fold change (FC) ≥ 2.0 between CRC and normal samples. Multiple hypothesis testing was controlled by applying the Benjamini–Hochberg false discovery rate (FDR) correction (Benjamini and Hochberg, 1995). Genes expressed in blood are identified using data from GTEx portal (https://gtexportal.org/home/). We implemented Venn diagram approach in order to find the genes that have CNAs with concomitant gene expression changes and are also expressed in blood. Our methodology is shown in Figure 1.

### Diagnostic validation of the gene signature

For validating the diagnostic and prognostic value of our gene signature, we used independently performed microarray and RNA sequencing datasets from The Cancer Genome Atlas (TCGA) database. The microarray data (TCGA data version 2016_01_28 for colorectal adenocarcinoma (COADREAD) included 244 samples (222 tumor and 22 normal samples) and the RNAseq data contained 675 samples (624 tumor and 51 normal). We used level 3 preprocessed and normalized gene expression data as described in detail by the TCGA workgroup (https://gdac.broadinstitute.org/). We performed unsupervised principal component analysis (PCA) and hierarchical clustering by Pearson correlation with average linkage clustering to validate the diagnostic performance of our gene signature. Moreover, transcription profiling datasets of blood samples from CRC patients (*n* = 100) and healthy controls (*n* = 100) were retrieved from ArrayExpress database (E-MTAB-1532) to test the gene signature expression levels in blood samples from patients with CRC as compared to those from normal controls.

### Colorectal cancer classifier model and performance evaluation

We designed a 17-gene-CRC classifier using several machine learning algorithms, including Support Vector Machine (SVM), K-Nearest Neighbor (KNN), Linear Discriminant Analysis (LDA), and Nearest Centroid. First, we used the GSE23878 dataset for building the classification model, and then tested the classification performance on an indepedent dataset (TCGA dataset) to confirm if the 17-gene-classifier can distinguish patients from normal controls. We evaluated the performance of the classifier for its accuracy, specificity, sensitivity, and area under curve (AUC), as described previously (Al-Harazi et al., 2021a; Al-Harazi et al., 2021b). The analyses were performed using PARTEK Genomics Suite (Partek Inc., St. Lois, MO, United States).

### Survival and multivariate analyses

We performed univariate and multivariate analyses using the Cox proportional hazard regression model to investigate the prognostic value of our gene signature along with other clinic-pathological variables. We defined a risk score for each patient in the TCGA dataset as a linear combination of expression level of 17 genes multiplied by the regression coefficient β) of each gene extracted from the Cox proportional hazards regression model, using the following formula: prognosis risk score = expression of gene1 × β1+ expression of gene2 × β2 + … expression of gene_n_ × *β*_n_. Patients are defined as high and low risk groups using the median score as the cutoff. We then used the Kaplan-Meier method to plot survival curves. Significance between survival curves was calculated by log-rank test. Univariate Cox regression analysis was performed to evaluate the prognostic value of the 17-gene signature and their relationships with overall survival of CRC patients. Moreover, multivariate Cox regression analysis was performed to examine the predictive ability of the 17-gene signature independent of other clinical factors, including gender, age, pathologic stage, and lymphatic invasion. A *p*-value < 0.05 was considered statistically significant.

### Gene ontology enrichment, canonical pathway, and network analyses

Functional, pathway, gene ontology (GO) enrichment, and gene interaction network analyses of the identified gene signature were performed using QIAGEN’s Ingenuity Pathway Analysis (IPA) (QIAGEN Inc., https://www.qiagenbioinformatics.com/products/ingenuity-pathway-analysis), DAVID bioinformatics tools (Sherman et al., 2007), and PANTHER™ classification systems (Thomas et al., 2003). We performed gene interaction network and causal network analyses after mapping the identified gene signature to its corresponding gene object in the Ingenuity pathway knowledge base. A right-tailed Fisher’s exact test was used to calculate a *p*-value determining the probability that the biological function (or pathway) assigned to the data set is explained by chance alone (Colak et al., 2020).

## Results

### Identification of a gene signature associated with colorectal cancer

**FIGURE 2:**
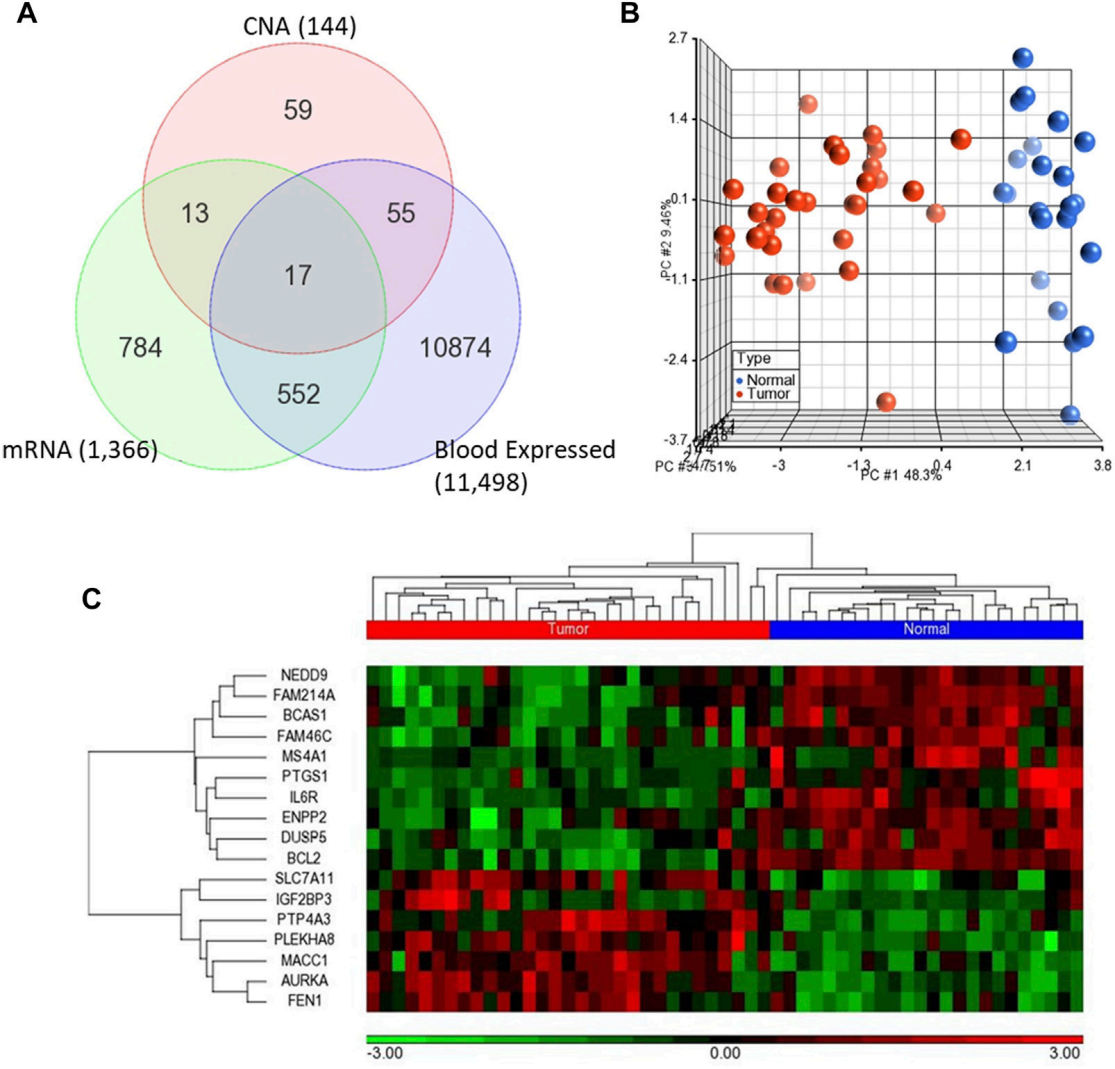
**(A)** Venn diagram representing the overlapping 17 genes among differentially expressed genes (mRNA) and CNA that are also expressed in blood **(B–C)** Unsupervised principal component analysis (PCA) and two-dimensional hierarchical clustering using 17-gene signature on the GSE23878. The red spheres refer to tumors and blue ones for normal controls. The hierarchical clustering resulted in two main clusters of tumors and controls. Samples are denoted in columns and genes are denoted in rows.

We first analyzed global mRNA expression profile of patients with CRC (*n* = 35) and normal controls (*n* = 24) using data from GSE23878 (Uddin et al., 2011). The analysis revealed 1,366 DEGs with adjusted *p*-value < 5% and absolute fold-change >2 in tumor compared to normal (Supplementary Table S1). Following that, we identified significantly dysregulated genes that have also copy number alterations (gains/losses) by mapping these dysregulated genes on the CNA regions in the genomic data from CRC patients and controls (Eldai et al., 2013). There were 144 genes in CNA regions of CRC patients. Of note, the patients in the transcriptomic and genomic data all belong to the same ethnicity (Arabs) (Eldai et al., 2013). Having ethnically matched cohorts in both types of omics measurements would limit the bias due to ethnicity and may reveal more biologically relevant results. Integrating with the genes in the CNA regions revealed that 30 of the significantly dysregulated genes have concomitant copy number alterations, 17 of which are also expressed in blood (Figure 2A; Table 1).

**TABLE 1:** The 17-gene signature that is identified in this study for CRC.

Gene	Gene title	*p*-value	FC
*AURKA*	aurora kinase A	3.62E-09	2.84
*BCAS1*	breast carcinoma amplified sequence 1	2.99E-07	−2.24
*BCL2*	B Cell CLL/lymphoma 2	3.95E-11	−2.92
*DUSP5*	dual specificity phosphatase 5	2.77E-06	−2.28
*ENPP2*	ectonucleotide pyrophosphatase/phosphodiesterase 2	2.98E-07	−2.61
*FAM214A*	family with sequence similarity 214, member A	1.13E-09	−2.09
*FAM46C*	family with sequence similarity 46, member C	1.45E-06	−2.29
*FEN1*	flap structure-specific endonuclease 1	3.50E-07	2.02
*IGF2BP3*	insulin-like growth factor 2 mRNA binding protein 3	0.010319	2.01
*IL6R*	interleukin 6 receptor	5.97E-10	−2.91
*MACC1*	metastasis associated in colon cancer 1	8.75E-09	3.87
*MS4A1*	membrane-spanning 4-domains, subfamily A, member 1	2.52E-07	−2.63
*NEDD9*	neural precursor cell expressed, developmentally down-regulated 9	2.21E-07	−2.18
*PLEKHA8*	pleckstrin homology domain containing, family A	3.40E-09	2.14
*PTGS1*	prostaglandin-endoperoxide synthase 1 (prostaglandin G/H synthase and cyclooxygenase)	7.25E-06	−2.07
*PTP4A3*	protein tyrosine phosphatase type IVA, member 3	2.38E-08	2.37
*SLC7A11*	solute carrier family 7 member 11	2.34E-07	2.89

* Abbreviation: FC, fold change; FC, is calculated between the mean expression value observed in tumor compared to normal. Negative (−) value indicates down-regulation.

### Validation of the 17-gene signature for diagnostic and prognostic potential

**FIGURE 3:**
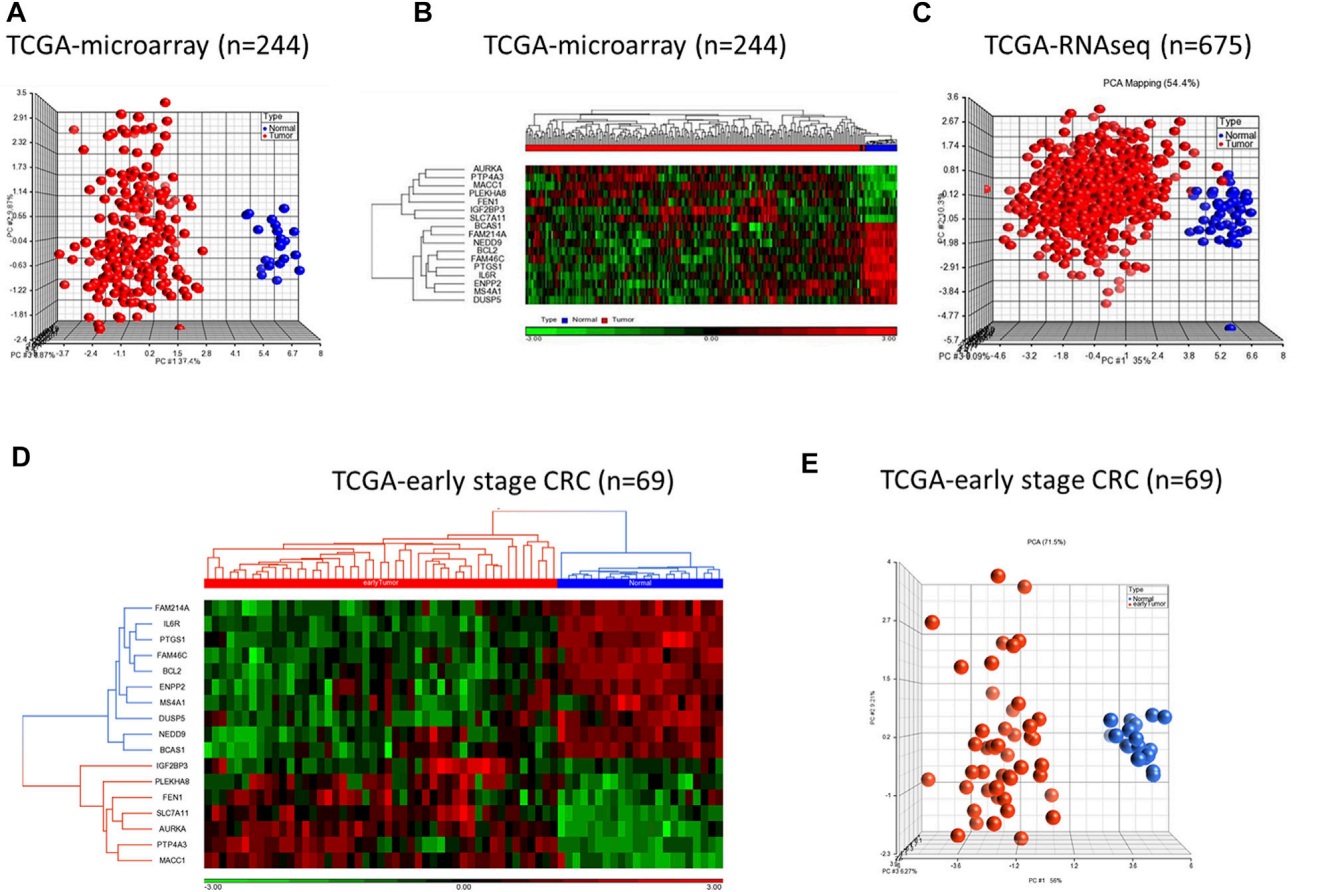
PCA and hierarchical clustering analyses using 17-gene signature on the TCGA microarray dataset (*n* = 244) **(A–B)** and TCGA RNA-sequencing dataset (*n* = 675) **(C)**. The hierarchical clustering and PCA analyses using the 17-gene signature on early stage CRC data from TCGA (*n* = 69) **(D–E).** The analyses clearly distinguished patients as either CRC or normal controls on all datasets. Red and blue indicate tumor and normal samples, respectively.

We validated diagnostic value of the 17-gene signature on GSE23878 (Figure 2) as well as on two independent datasets; TCGA microarray (*n* = 244 samples) and TCGA RNA-sequencing datasets (*n* = 675 samples) (Figures 3A–C, respectively). The unsupervised PCA and two-dimensional hierarchical clustering clearly distinguished patients as either CRC or normal controls in all datasets (Figures 2B, C, 3A–C). We also used early stage CRC data from TCGA (*n* = 47, Stage I tumor) to test the 17-gene signature’s diagnostic potential to discriminate the early stage CRC patients from normal controls. The analysis provided 100% accurate clustering of the two groups (Figures 3D, E). Moreover, we investigated our 17-gene signature’s expression level within blood samples obtained from CRC patients (*n* = 100) and healthy controls (*n* = 100) (E-MTAB-1532), which revealed that those with CRC have significantly higher expression levels than the controls (*p*-value <0.0001) (Supplementary Figure S1).

**FIGURE 4:**
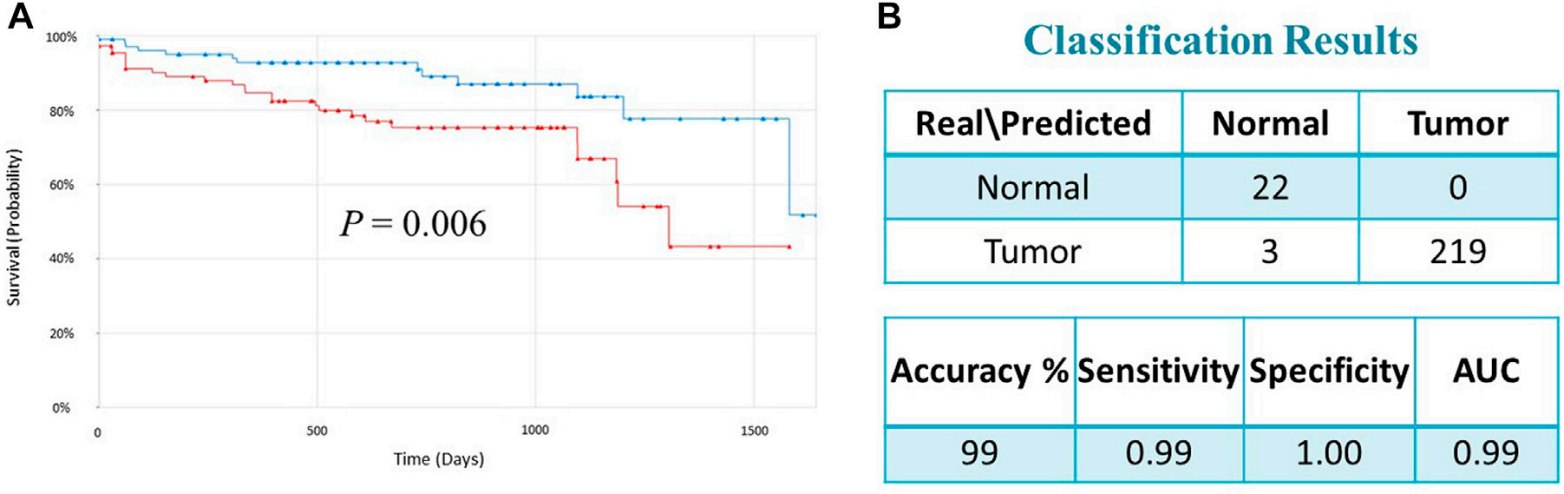
**(A)** Kaplan–Meier survival analysis of the TCGA dataset indicated that the high-risk group had significantly worse prognosis than the low-risk group (*p* = 0.006). Red and green curves indicate high and low-risk groups, respectively. **(B)** Classification performance of the 17-gene classifier modeled using SVM with linear kernel algorithm. The classification performance is evaluated on the TCGA dataset (*n* = 244).

To validate the prognostic significance of the 17-gene signature, we used the TCGA dataset with detailed clinical information and overall survival. We first calculated a prognostic risk score based on 17-gene signature, as described in the methods section and patients are classified as high or low risk using the median score as a cutoff. Our results demonstrated that a high 17-gene prognostic score is significantly associated with poor disease outcome (*p*-value = 0.006). Indeed, Kaplan–Meier survival analysis displayed that the high-risk group had significantly worse prognosis than the low-risk group (Figure 4A). Furthermore, the multivariate Cox regression analysis revealed that 17-gene signature prognosticated the CRC outcome independent of other clinical variables, including age, gender, pathologic stage and lymphatic invasion (HR = 2.61, 95% CI = 1.3–5.23; *p* = 0.0069) (Table 2).

**TABLE 2:** Univariate and multivariate analysis associated with CRC overall survival.

Variables	Univariate analysis		Multivariate analysis	
	*p*-value	HR (95% CI)	*p*-value	HR (95% CI)
Age (years)				
≥50 vs. < 50	0.73	0.81 (0.25–2.66)	0.76	1.21 (0.36–4.13)
Gender				
Female vs. Male	0.22	1.49 (0.79–2.81)	0.73	1.12 (0.58–2.19)
Pathologic Stage				
III-IV vs. I-II	**0.0003**	3.45 (1.78–6.70)	**0.005**	2.97 (1.39–6.4)
Lymphatic Invasion				
Yes vs. No	**0.004**	2.78 (1.38–5.62)	0.37	1.44 (0.65–3.18)
Risk Score				
High vs. Low	**0.007**	2.56 (1.29–5.1)	**0.0069**	2.61 (1.3–5.23)

Bold indicates significance. Abbreviations: CI, confidence interval; HR, hazard ratio.

### Classification model and performance assessment

We designed a 17-gene CRC classifier using different classification algorithms, including Support Vector Machine (SVM), K-Nearest Neighbor (KNN), Linear Discriminant Analysis (LDA), and Nearest Centroid and estimated the classification performance. The GSE23878 dataset is used for building the classification model and the classification performance is tested on an indepedent dataset (TCGA dataset). We assessed the classifier’s performance in terms of accuracy, specificity, sensitivity, and area under the curve (AUC), as described previously (Al-Harazi et al., 2021a; Al-Harazi et al., 2021b). The SVM with linear kernel has outperformed other algorithms and the 17-gene classifier achieved a high accuracy of 99 percent, and sensitivity, specificity and AUC of 99%, 100% and 99%, respectively (Figure 4B), confirming the 17-gene signature’s ability to discriminate patients from normal controls.

### Functional, pathway and gene interaction network analyses

**FIGURE 5:**
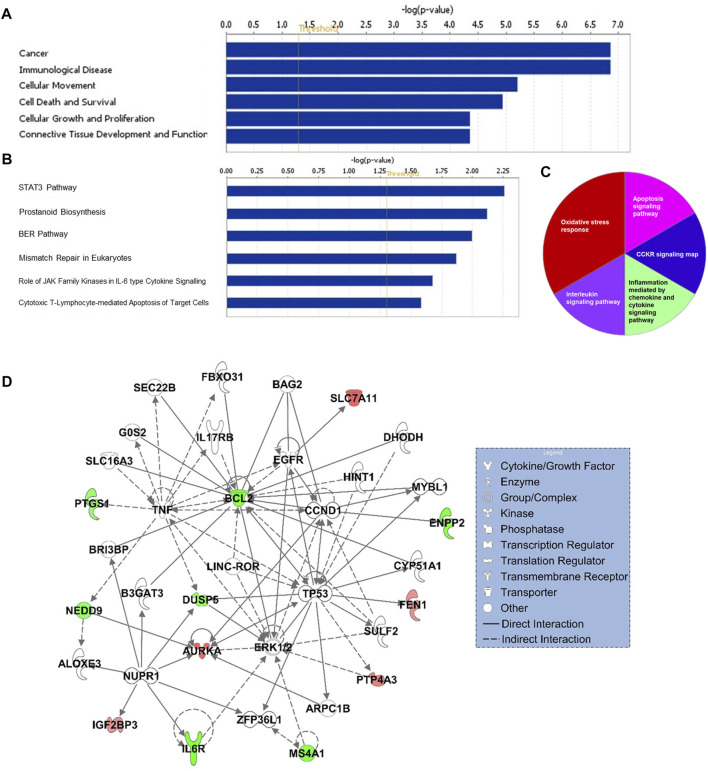
Functional **(A)** and canonical pathway **(B)** and PANTHER pathway **(C)** analyses of 17-gene signature. *X*-axis (in A and B) represents–log (*p*-value); the significance of the functional/pathway term. The threshold line indicates *p*-value of 0.05. **(D)** Gene interaction network analyses of 17-gene signature. Red/green indicates higher/lower expression in CRC in comparison to controls.

Gene ontology enrichment and functional analyses revealed that the 17-gene signature is significantly associated with diseases and functions related to cancer, cellular movement, cellular growth and proliferation, cell death and survival (Figure 5A and Supplementary Table S2). Moreover, pathway analysis using several bioinformatics tools revealed alterations in STAT3, ERK/MAPK, oxidative stress, interleukin and cytokine signaling al pathways (Figures 5B,C). Gene interaction network analysis indicated hub genes that may have potentially important role in CRC transformation and progression, including *BCL2, MS4A1, AURKA, IL6R, TP53, NUPR1, DUSP5,* and *CCND1* (Figure 5D). Furthermore, IPA causal network analyses revealed predicted activation of *DICER1* and *SMAD3* in CRC (Supplementary Figure S2).

## Discussion

In this study, we aimed to identify a robust gene signature that would detect the disease accurately and have prognostic significance that would differentiate the high-risk patients from the low-risk ones. Since appropriate management of choice and curative surgical resection success rate depend largely on staging of the cancer, convenient and non-invasive early detection biomarkers are still needed to ensure early diagnosis and good prognosis (Al Bandar and Kim, 2017; Feo et al., 2017).

We performed an integrated analysis of significantly dysregulated genes within the transcriptome of CRC patients with the genes that have copy number changes (gains/losses) in a patient cohort from an ethnically matched population, and identified a blood-based 17-gene signature. We then validated its diagnostic and prognostic potential on an independent large cohort of CRC patients. Previous studies have demonstrated that multi-omics analysis (using whole-genome gene expression profiling, copy number variations (CNVs), proteomics, metabolomics, and others) may lead to reliable biomarkers that are robust in disease classification and may also help identify cancer driver genes that are involved in tumor initiation and progression (Colak et al., 2010; Colak et al., 2013; Ohshima et al., 2017; Liu et al., 2021; Kaya et al., 2022; Ruan et al., 2022). Moreover, integrating omics data with the gene interaction networks has been shown to be a robust methodology that may lead to more reliable and accurate predictive biomarkers for human diseases (Al-Harazi et al., 2016; Ma et al., 2019; Khan et al., 2020; Seifert et al., 2020; Sinkala et al., 2020).

Our gene network analysis revealed several key hub genes that may have potentially important roles in CRC transformation and progression, including *BCL2* (Lindner et al., 2017; Perini et al., 2018; Diaz-Flores et al., 2019), *MS4A1* (Mudd et al., 2021; Li and Fang, 2022)*, AURKA* (Wang et al., 2020; Mou et al., 2021; Kahl et al., 2022)*, IL6R* (Mendez-Clemente et al., 2022)*, TP53* (Oner et al., 2018), *NUPR1* (Martin et al., 2021; Xiao et al., 2022), *DICER1* (Iliou et al., 2014; Luan et al., 2021), *DUSP5*, *SMAD3* (De Mattia et al., 2021; Tang et al., 2022), and *CCND1* (Shan et al., 2017; Chen et al., 2020). Some of the identified genes were reported to be associated with cancers, including colorectal cancer. For example, *BCL2* family are central regulators of apoptosis, and up-regulation of *BCL2* has been shown to lead to tumor development and progression as well as resistance to cancer therapy (Lindner et al., 2017; Perini et al., 2018; Diaz-Flores et al., 2019). *MS4A1* encodes a B-lymphocyte surface molecule CD20 that has been reported to be associated with lipid metabolism and immune cell activation, and its expression is an independent predictor of cancer prognosis (Mudd et al., 2021; Li and Fang, 2022). Aurora kinases are involved in cell cycle regulation, G2/M transition, mitosis, and DNA replication functions. Recent reports have shown that aurora kinase A (*AURKA*), *IL6R*, *NUPR1*, and *DICER1* play important role in the development, progression, and metastasis of a variety of cancers including colon cancer (Iliou et al., 2014; Wang et al., 2020; Luan et al., 2021; Martin et al., 2021; Mou et al., 2021; Kahl et al., 2022).

The causal network analysis indicated predicted activation of *DICER1* and *SMAD3* in CRC. Recent studies have shown that *DICER1* is involved in the cancer initiation and development (Ma et al., 2020; Luan et al., 2021). Although the underlying mechanism is still unclear, transfer RNA-derived fragment biogenesis by *DICER1* is directly associated with cancer development. A high expression of the enzyme is related to poor survival, independent of the patient’s other predisposing factors (Luan et al., 2021). SMAD3 has also been shown to be associated with tumor initiation and progression in earlier studies in several cancers (Colak et al., 2010; De Mattia et al., 2021; Tang et al., 2022). It has been also reported to have tumor promotor roles and directly involved in epithelium to mesenchyme transition (EMT), hence enhancing invasion, migration and metastasis (Millet and Zhang, 2007).

The pathway analyses indicated significant alterations in several cancer-related signaling pathways, such as oxidative stress, STAT3, ERK/MAPK, interleukin and cytokine signaling pathways. The ERK enzyme belongs to the MAPK family, which is involved in a various signaling cascades that regulate fundamental cellular processes such as cell growth, proliferation, differentiation, as well as stress responses. Our study as well as prior research findings have shown that there is a strong correlation with MAPK inhibition, especially ERK inhibition, and the development and advancement of most cancer types. ERK pathway dysfunction plays a major role in tumor invasion and metastasis, with varying level of different components of the cascade depending on the type of cancer. This makes it an abundant oncogenic factor that can also be used to identify CRC and differentiate it from other tumors (Guo et al., 2020).

In conclusion, the 17-gene signature that is identified in this study revealed genes and pathways that may be critical for CRC transformation and progression, and has the potential to detect the disease non-invasively as well as predict its outcome.

## Data Availability

The datasets used in this study can be found in online repositories, including The Cancer Genome Atlas (TCGA), ArrayExpress, and the NCBI Gene Expression Omnibus (GEO). The names of the repositories and accession numbers can be found in this article/materials and methods.
