# Supplementary material for: Transcriptomic data analysis coupled with copy number aberrations reveals a blood-based 17-gene signature for diagnosis and prognosis of patients with colorectal cancer
Source: Front Genet. 2023 Jan 6;13:1031086. doi: 10.3389/fgene.2022.1031086 (PMC9854115; doi:10.3389/fgene.2022.1031086)

**Supplementary Table 2. Gene ontology enrichment analysis of the 17-gene signature**

| GO Biological Process | Count | % | P-Value | Genes |
| --- | --- | --- | --- | --- |
| regulation of cellular protein metabolic process | 6 | 35.29 | 2.67E-02 | DUSP5, ENPP2, BCL2, AURKA, IGF2BP3, IL6R |
| cell migration | 5 | 29.41 | 1.08E-02 | PTP4A3, ENPP2, BCL2, IL6R, SLC7A11 |
| localization of cell | 5 | 29.41 | 1.61E-02 | PTP4A3, ENPP2, BCL2, IL6R, SLC7A11 |
| cell motility | 5 | 29.41 | 1.61E-02 | PTP4A3, ENPP2, BCL2, IL6R, SLC7A11 |
| positive regulation of cellular protein metabolic process | 5 | 29.41 | 2.01E-02 | DUSP5, ENPP2, BCL2, AURKA, IL6R |
| positive regulation of protein metabolic process | 5 | 29.41 | 2.46E-02 | DUSP5, ENPP2, BCL2, AURKA, IL6R |
| locomotion | 5 | 29.41 | 2.58E-02 | PTP4A3, ENPP2, BCL2, IL6R, SLC7A11 |
| positive regulation of phosphorylation | 4 | 23.53 | 3.28E-02 | DUSP5, ENPP2, BCL2, IL6R |
| protein dephosphorylation | 3 | 17.65 | 1.64E-02 | DUSP5, PTP4A3, BCL2 |
| response to oxidative stress | 3 | 17.65 | 3.67E-02 | BCL2, PTGS1, SLC7A11 |

**
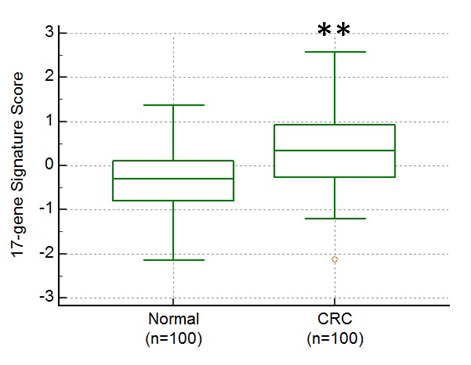
Supplementary Figure 1.** The 17-gene signature average expression scores^a^ in CRC samples (n=100) are significantly higher than the ones in controls (n=100)

Abbreviations: ^a^The 17-gene signature expression score is defined as the average expression of up-regulated genes $-$ average expression of down-regulated genes for each sample. (**, P-value < 0.0001).

**Supplementary Figure 2.** IPA causal network analyses after mapping the identified gene signature to its corresponding gene object in the Ingenuity pathway knowledge base.


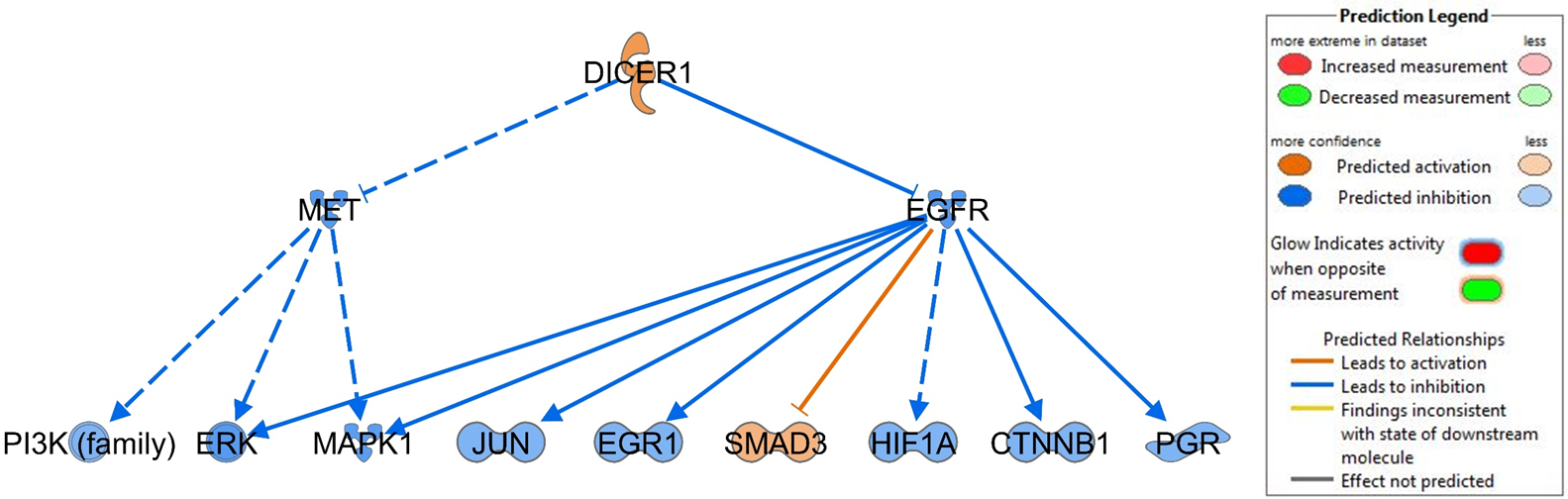

Supplement: Supplementary file 2 [file DataSheet1.docx]
